# From the scala naturae to the symbiogenetic and dynamic tree of life

**DOI:** 10.1186/1745-6150-6-33

**Published:** 2011-06-30

**Authors:** Ulrich Kutschera

**Affiliations:** 1 Institute of Biology, University of Kassel, Heinrich-Plett-Str. 40, D-34109 Kassel, Germany

## Abstract

All living beings on Earth, from bacteria to humans, are connected through descent from common ancestors and represent the summation of their corresponding, ca. 3500 million year long evolutionary history. However, the evolution of phenotypic features is not predictable, and biologists no longer use terms such as "primitive" or "perfect organisms". Despite these insights, the Bible-based concept of the so-called "ladder of life" or *Scala Naturae*, i.e., the idea that all living beings can be viewed as representing various degrees of "perfection", with humans at the very top of this biological hierarchy, was popular among naturalists until ca. 1850 (Charles Bonnet, Jean Lamarck and others). Charles Darwin is usually credited with the establishment of a branched evolutionary "Tree of Life". This insight of 1859 was based on his now firmly corroborated proposals of common ancestry and natural selection. In this article I argue that Darwin was still influenced by "ladder thinking", a theological view that prevailed throughout the 19th century and is also part of Ernst Haeckel's famous Oak tree (of Life) of 1866, which is, like Darwin's scheme, static. In 1910, Constantin Mereschkowsky proposed an alternative, "anti-selectionist" concept of biological evolution, which became known as the symbiogenesis-theory. According to the symbiogenesis-scenario, eukaryotic cells evolved on a static Earth from archaic prokaryotes via the fusion and subsequent cooperation of certain microbes. In 1929, Alfred Wegener published his theory of continental drift, which was later corroborated, modified and extended. The resulting theory of plate tectonics is now the principal organizing concept of geology. Over millions of years, plate tectonics and hence the "dynamic Earth" has caused destructive volcanic eruptions and earthquakes. At the same time, it created mountain ranges, deep oceans, novel freshwater habitats, and deserts. As a result, these geologic processes destroyed numerous populations of organisms, and produced the environmental conditions for new species of animals, plants and microbes to adapt and evolve. In this article I propose a tree-like "symbiogenesis, natural selection, and dynamic Earth (synade)-model" of macroevolution that is based on these novel facts and data.

**Reviewers:**

This article was reviewed by Mark Ragan, W. Ford Doolittle, and Staffan Müller-Wille.

## Background

In his *Autobiography *[1], Charles Darwin (1809 - 1882) presented a self-critical review of his achievements as a naturalist that revealed much about the character of this key figure of the evolutionary sciences and other branches of biology and geology [2-4]. With respect to the most influential of Darwin's 16 scientific books, *On the Origin of Species*, the author remarked that "Sixteen thousand copies have now (1876) been sold in England and considering how stiff a book it is, this is a large sale" [1]. This judgement is in part due to the fact that the *Origin of Species *was not designed by Darwin as a separate book; rather, it was published as an *Abstract*, taken from a much larger manuscript entitled *Natural Selection *[5]. Ironically, Darwin's major, scheduled "Magnum opus" with the tentative title *Natural Selection *never appeared in print, but the *Extract *published by the author in November 1859 in order to establish priority with respect to his theory of the "preservation of favourable variations and the rejection of injurious variations" became a best- and longseller [6].

The second and more important reason for the "stiffness" of Darwin's *Origin of Species *is attributable to the almost complete lack of illustrations. In contrast to Darwin's books on botanical and zoological issues, which contain numerous pictures [2-4], his *Abstract *published in 1859 (6th and final edition, 1872) [6,7] contained only one rather "sterile" diagram, a phylogenetic scheme. This "tree-like" figure is part of Chapter IV entitled "Natural Selection" in the first edition [6], and re-named "Natural Selection; or the Survival of the Fittest" in the 6th and final version of the "Species book" [7]. It should be noted that the phrase "survival of the fittest" was borrowed by Darwin from the philosopher Herbert Spencer (1820-1903), who was also the first to introduce the word "evolution" *sensu *phylogenetic development (a term not used by Darwin in the first edition [6]) into the emerging biological sciences of the 19th century [5].

With reference to his abstract illustration, Darwin explained at length the principle of "descent with modification by means of natural selection", and concluded, with his Bible-educated readers in mind, that "On the view that each species has been independently created, I can see no explanation of this great fact (i.e., the relatedness of all animals and all plants) in the classification of all organic beings; but, ..., it is explained though inheritance and the complex action of natural selection, entailing extinction and divergence of character as we have seen illustrated in the diagram" [6] p. 100.

Although Darwin made many changes and added entire sections to the text during the five revisions of his original version of the *Origin *[6], one key sentence remained unchanged: At the end of Chapter IV, the author wrote, with reference to his tree-like diagram, that "As buds give rise by growth to fresh buds, and these, if vigorous, branch out and overtop on all sides many a feebler branch, so by generation I believe it has been with the great Tree of Life, which fills with its dead and broken branches the crust of the earth, and covers the surface with its ever-branching and beautiful ramifications" [6], p. 101; [7], p. 137.

In this article I argue that the metaphorical "Tree of Life"-statement quoted above was still heavily rooted in the religious pre-Darwinian "evolutionary ladder-" or *Scala Naturae*-thinking of earlier naturalists. In the second part of this account I show that Darwin's view of a static, "Animals and Plants-based Tree of Life" that does not take into account micro-organisms and endosymbiotic events, is outdated. Finally, I review evidence indicating that the dynamic Earth (plate tectonics), geological processes unknown to Darwin, must be integrated into a more realistic picture of the evolution of life on our ever changing "planet of the microbes" [5].

## From the earliest Moral Tree to the Great Chain of Being

**Figure 1:**
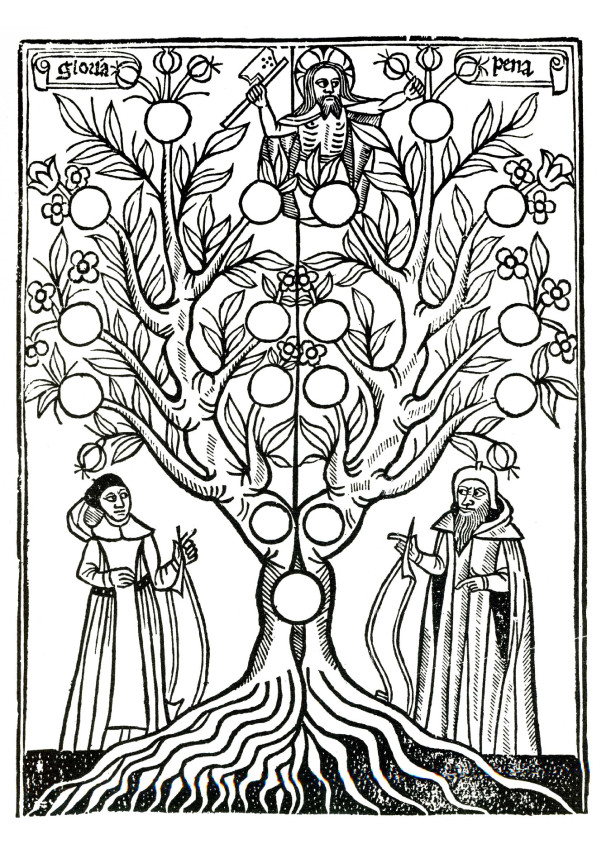
**The "Moral Tree", published posthumously in 1505 in a book authored by Raymon Llull (1232-1315)**. This Spanish philosopher and Christian theologian mixed up natural phenomena with supernatural religious dogma and hence became the spiritual father of a medieval ideology called "Llullism" [part of a woodcut, adapted from ref. 8].

The Spanish philosopher and theologian Ramon Llull (1232-1315) was one of the first to publish a tree-like scheme illustrating the growth and interrelationships of the basic knowledge of his time. Born into a wealthy family in Palma, and well educated, he worked as a teacher in Majorca and Paris. Llull's diagram of the "apostolic and moral tree" (Figure 1) formed part of a unified system of knowledge. At the top of this extensively rooted tree, Jesus, the incarnation of the Biblical God, is depicted, surrounded by the latin words "gloria" (fame) and "pena" (penalty). The woodcut depicted here is a modified version of the original that does not show all the details [8]. In his writings, Ramon Llull argued that there is no difference between philosophy (i.e., natural history) and Bible-based theology, and therefore between reason and faith [8,9]. Hence, even the most absurd mysteries may be proven by means of logical inferences and the use of Llull's *Ars Magna *[9]. This way of thinking removed all distinctions between natural (fact-based) truths and supernatural (spiritual) myths.

This "rationalistic mysticism" was taken up by the followers of the Spanish theo-philosopher ("Llullists") and later evolved into an ideology that was called "Llullism" [9]. The basic tenet of the "Illuminated Doctor" is illustrated in his apostolic tree, which depicts real things (humans and a tree-like plant), mixed up with a supernatural being (Jesus, the son of God, as the crown of the "tree of knowledge") [8]. Since Llull also wrote treatises on medieval natural history (alchemy, botany), and had a great influence on the mathematician Gottfried W. Leibnitz (1646-1716), he is also recognized as a pioneer in computation theory. However, since he had several religious visions, and was a convinced Christian, Raymon Llull, throughout his later life, mixed up facts of nature and religious imaginations [9].

Although the influence of the "Llullists" may have been limited, the pre-Christian idea of the *Scala Naturae *("Great Chain of Being") [10-14] is unequivocally related to the Bible-based scheme depicted in Figure 1. The order of the static world, between "earth and heaven", was shown and thought of as a linear sequence of bodies (from minerals via plants, animals to man). On top of this hierarchical arrangement of "created beings" we find the almighty Biblical God, who, according to Llull, had the following positive attributes: "goodness, greatness, power, eternity, wisdom, will, virtue, truth, and glory" [9].

**Figure 2:**
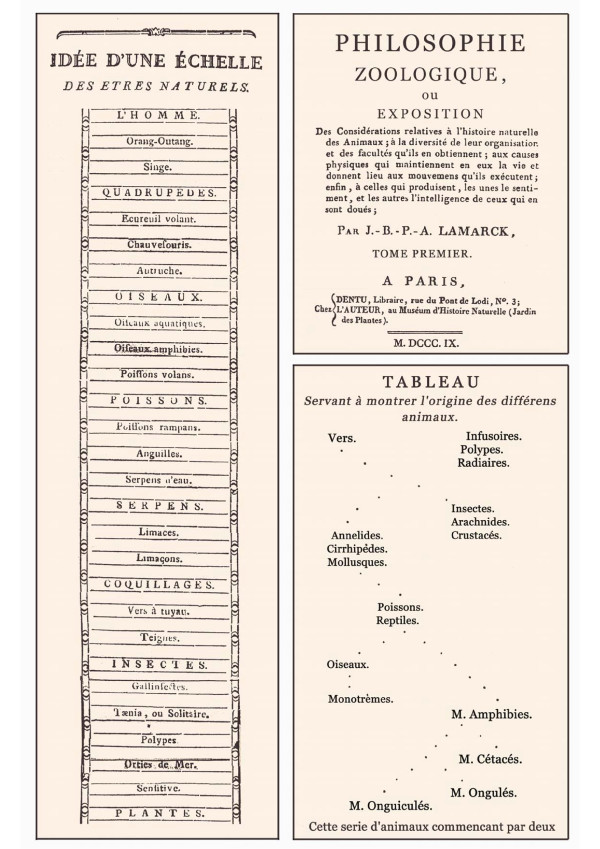
**Upper part of the "Great Chain of Being" or *Scala Naturae*, as published in 1745 by Charles Bonnet (1720-1793) (left column)**. On the right side, the title page of the most influential book of Jean Lamarck (1744-1829) and his tree-like scheme of 1809 is shown [adapted from refs. 15 and 16].

One popular version of the *Scala Naturae*, which was published in 1779 by the Swiss naturalist Charles Bonnet (1720-1793), is shown in Figure 2. At the base of this version of the "Great Chain of Being" are non-living objects such as minerals and earth, followed by plants, insects, reptiles (snakes) and mammals. On top of this "natural ladder" we find the "Orang-Outang", followed by "L'Homme" (man) [15]. It should be noted that Bonnet, who discovered the phenomenon of parthenogenesis in insects, was convinced that species do not change over long time periods. In his monograph of 1779 from which the *Scala Naturae *is reproduced [15], Bonnet concluded that there is no visible change in nature, everything remains largely identical, and species are constant. Hence, Bonnet and most naturalists of his time were convinced that animals and plants are static essences created individually by the Biblical God, a religious view that Darwin attacked and thoroughly refuted [5-7,11-14].

## Jean Lamarck and the origin of evolutionary tree-thinking

The French botanist and zoologist Jean-Baptiste de Lamarck (1744-1829) was expected by his father to take a career in the Catholic church. However, the young naturalist was not inclined to the ministry, and after his father's death in 1860 Lamarck quit his Jesuit college to become a botanist. Later, he made the switch to zoology, coined key-terms such as "invertebrates", and published new concepts on the relationships between different groups of animals [16,17].

In his most important book entitled *Philosophie Zoologique *[16], Lamarck juxtaposed the "conventional view", i.e., the dogma of independent creations of animals (and plants) as described in the Bible, with his "new opinion": According to Lamarck [16], there are ongoing spontaneous generations of primitive forms of life on Earth. Later, these non-specialized living beings transformed into "higher" animals during the history of our planet. Hence, fifty years before Darwin's *Origin of Species *was published [6], Lamarck proposed, in 1809, the principle of the gradual transformation of species and depicted his "theory of evolution" in a famous tree-like diagram that is reproduced in Figure 2.

However, a comparison of Lamarck's original scheme with Bonnet's *Scala Naturae *reveals striking similarities: The "Tableau" of Lamarck, who was an adherent of the philosophy behind the "Great Chain of Being" [14], is more "ladder-like" than a true tree with branches and twigs (Figure 2). In a subsequent book published in 1815, Lamarck depicted an "Order presuming the formation of animals in two separate series" [17]. This scheme [reproduced in ref. 14] is again more a ladder than a tree. The author distinguished between three hierarchy levels: Apathic-, sensible- and intelligent animals, respectively.

Concerning the means by which the structure of an organism altered over generations, Lamarck proposed his famous theory that is still known today as the "inheritance of acquired characteristics". According to the French scientist, changes occurred because an animal passed on to its offspring physiological changes, such as strengthened muscules it had acquired in its own lifetime, and those modifications came about in response to its survival needs. Conversely, the disuse of an organ would cause it to wither and disappear, which "explained", how snakes lost their legs etc. It should be noted that Lamarck's concept of inheritance, which is not supported by empirical evidence [18], was accepted by Darwin. In addition, Lamarck suggested that species transformations happen according to a pre-determined plan and that the results have already been decided by forces he was unable to identify.

Although Lamarck's theory of the gradual transmutation of species over long (geological) time periods was popular until his death in 1829, his ideas encountered fierce religious and political opposition, notably by Georges Cuvier (1769-1832). As a result, the achievements of Lamarck were soon forgotten so that his "principle of the gradual transformation of species" was superseded again by Biblical myths.

## Charles Darwin's Tree of Life and the sterile, static hierarchy of nature

In a little-known paper of 1855 entitled "On the law which has regulated the introduction of new species" [19], Alfred Russel Wallace (1823-1913), the co-discoverer of the Darwinian "principle of natural selection" [20], described a "Tree of Life-concept" referring to "branching of the lines of affinity, as intricate as the twigs of a gnarled oak ... and to ..... minute twigs and scattered leaves". In an article published one year later, Wallace described a method of tree-building, which has recently been discussed in this journal [14].

**Figure 3:**
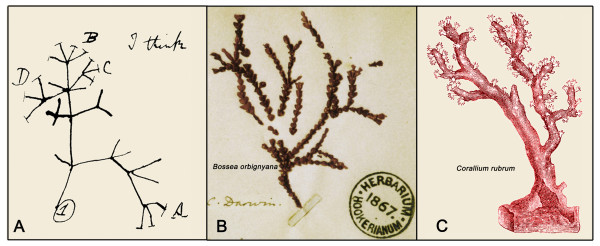
**Charles Darwin's early sketch of an evolutionary tree (or a coral), drawn in 1837 **(A). Marine organism (*Bossea arbignyana*) collected by Darwin and classified by him as a coral ("family Corallinae"). Later it was discovered that this "coral-like" inhabitant of sea waters (B) is a red alga (family Corallinaceae, Phylum Rhodophyta). Red coral (*Corallium rubrum*), which grows on rocky sea bottom either in the depths or in dark caverns (C). This wide-spread species, which is found mainly in the Mediterranean Sea, was known to Darwin and possibly served as a model for his diagram (see Figure 4) [adapted from ref. 22].

Charles Darwin's famous first "Tree of Life"-sketch, which was supplemented by the phrase "I think" (Figure 3A), was drawn into his "Notebook B" of 1837, only one year after the junior scientist had returned from his five-year long voyage on *HMS Beagle *[5]. Darwin's sketch appears on page 36 of his "Notebook B" - the first 35 pages are taken up by considerations on the "evolutionary thoughts" of his famous grandfather Erasmus Darwin (1731-1802). The older Darwin published his revolutionary thoughts on the transformation of species in his book entitled *Zoonomia *(1794). With respect to (endothermic) mammals Erasmus Darwin wrote that "... would it be too bold to imagine that, in the great length of time since the earth began to exist, perhaps millions of years ... that all warm-blooded animals have arisen from one living filament, which the great First Cause endued with animality, ... and thus possessing the faculty of continuing to improve by its own inherent activity, and of delivering down those improvements by generation to its posterity, world without end? ... as the earth and ocean were probably peopled with vegetable productions long before the existence of animals ... shall we conjecture that one and the same kind of living filament is and has been the cause of all organic life?" [21].

It has been argued that, with respect to Charles Darwin's botanical works, the influence of his grandfather may have been larger than he later admitted [3]. The passage cited above suggests that the younger Darwin developed his famous "I think-sketch" of 1837 (Figure 3 A), at least in part, under the spiritual leadership of his grandfather Erasmus.

However, what is certain is that for Charles Darwin the "Tree of Life" was not so much thought of as a woody plant, but rather as a coral. As Bredekamp [22] has documented in detail, Darwin wrote in his "Notebook B" of 1837 that "The tree of life should perhaps be called the coral of life". This view of the "unity of life on Earth" was in part based on Darwin's hands-on experience as a geologist, who had studied coral reefs in nature. A coral-like organism, later identified as an red alga (*Bossea arbignyana*), was collected and preserved by Darwin (Figure 3 B). This living being resembles the wide-spread stone coral (*Corallium rubrum*) (Figure 3 C), which served as the living model organism for Darwin's novel concept that has been summarized under the term "tree-thinking" [23-25].

**Figure 4:**
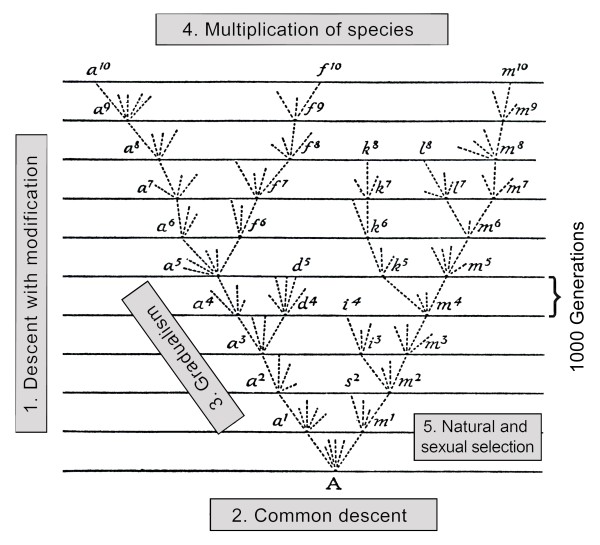
**Partial reproduction of the single illustration in Darwin's *Origin of Species *of 1859 (6. ed. 1872)**. This famous diagram may have been inspired by corals (or coral-like organisms) as depicted in Figure 3 B, C. Darwin's five theories are added to the figure (1. to 5.), which illustrate the transformation and diversification of species, which originate from a common ancestor (A) [adapted from ref. 7].

The only diagram Darwin included into the "sterile" text of his *Origin of Species *illustrates the essence of the "one long argument" developed by the author [6,7]. However, a comparison between the first and last (definitive) editions of 1859 and 1872, respectively, reveals a striking improvement of the text: Darwin (1872) had added a headline entitled "The probable effects of the action of natural selection through divergence of character and extinction, on the descendants of a common ancestor" to the text so that his discussion of the tree-like diagram became a separate paragraph in Chapter IV. On these pages of the *Origin*, Darwin's five "species theories" that were identified and described for the first time by Ernst Mayr (1904-2005) [11,12], are apparent (Figure 4):

1. Descent with modification (Darwin's definition of evolution) as a fact of nature *versus *supernatural acts of independent species creations, labelled by the author as religious dogma. 2. The principle of the last common ancestor of all forms of life (see A in Figure 4). 3. The theory of gradual, step-by-step species transformations. 4. The multiplication of species over evolutionary time (thousands of generations) and 5. The principles of natural (and sexual) selection as the major "driving forces" for the transformation of species.

In addition to these five "Darwinian species theories" [for details, see refs. 5, 11, 12], the author discussed the phenomenon of extinction. According to Darwin, the "improved descendant" of any species has the tendency to supplant and finally exterminate at each stage of evolutionary development "their predecessors and original progenitor" [6,7]. Finally, it should be noted that Darwin [6,7] unequivocally proposed a continuum between "speciation" and the evolutionary development of novel body plans, processes that were later called "micro- and macroevolution", respectively [11,12]. This concept was described by the British naturalist, with reference to his famous scheme (Figure 4), in the following words: "In the diagram, each horizontal line has hitherto been supposed to represent a thousand generations; but each may represent a million or more generations ... I see no reason to limit the process of modification ... to the formation of genera alone ... new families, or orders, are descended from two species of the original genus" [7].

Despite these tremendous insights provided by Darwin, who was one of the first to replace the "Ladder of Life" (Figure 2) by a "Tree-like concept" [22-25], our modern view of the biosphere, and the processes that have brought about the diversity of life as we know it today, have advanced to such an extent that Lamarck and Darwin would hardly understand our current evolutionary concepts. What are the problems with Darwin's 19th century-ideas about the evolution of life?

First, Darwin [6,7] used old-fashioned terms such as "perfection, improvement, higher vs. lower (or primitive) forms of life" etc. that are no longer in use today and may document relicts of religiously motivated "ladder-thinking" in his texts. Second, Darwin discussed in none of his 16 books in any detail the bacteria, although microbes were already known at that time [2]. In other words, the scientific work of the British naturalist is restricted to macro-organisms (animals, plants) to the exclusion of microbes. His references to "Infusoria", "Animalcules", or "Lower Organisms" [6,7] are confusing and unclear. Finally, although Darwin experienced a severe earthquake during his voyage with the *HMS Beagle *[5], his "species book" [6,7] is based on the implicit assumption that the Earth is a static planet.

Today it is well established by numerous independent studies that (1.) bacteria are, based on their collective protoplasmic biomass, the dominant forms of life, and by no means "primitive", (2.) endosymbiotic processes due to the fusion of ancient microbes have been key events in the history of life, and (3.) the Earth is not static, but dynamic. Our post-Darwinian view of the symbiogenetic and dynamic tree of life is described in the next sections.

## Ernst Haeckel's static trees and the origin of Monerology

In Germany, the zoologist Ernst Haeckel (1834-1919) was one of the most prominent popularizers of Darwin's ideas, notably of his "theory of descent with modification by means of natural selection" (i.e., the concepts 1. and 5. depicted in Figure 4). It should be noted that, in contrast to many of his colleagues, Haeckel fully acknowledged the achievements of Jean Lamarck. In one of his popular books, Haeckel argued that the term "Lamarckism" should be used to denote the principle of the transformation of species (i.e., evolution as such, corresponding to Darwin's theory no. 1), whereas the word "Darwinism" should denote the concept of natural selection, one of the British biologist's most important insights and contributions to the developing evolutionary sciences of the 19th century [20,26].

**Figure 5:**
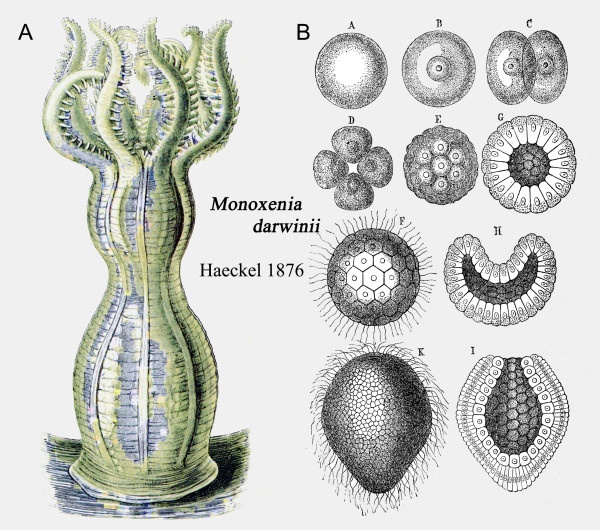
**Adult specimen of "Darwin's coral" and ontogenesis of Haeckel's model system.** Morphology of the coral *Monoxenia darwinii *(A), an organism discovered by Ernst Haeckel in 1873 in the Red Sea and later described by him as a new species, named in honour of Charles Darwin. The development of *M. darwinii *is shown in (B), from the fertilized egg (*A/B*) to the so-called "Becherlarve" (beaker larvae) or gastrula (*K/I*). Haeckel coined the term "Gastraea" to denote this phylogenetically conserved stage in animal development [adapted from ref. 27].

With respect to the "Tree of Life" as a coral-like structure (Figures 3 and 4) it should be remembered that Haeckel discovered and later described a marine coral from the Red Sea that was named after his friend and colleague Charles Darwin. A drawing of this organism (*Monoxenia darwinii *Haeckel 1876) is shown in Figure 5 A. One of Haeckel's greatest and most original contributions to evolutionary biology, his "Gastraea-Theorie", was based on his detailed investigations of the development of "Darwin's coral" (*Monoxenia darwinii*). In a journal article published in 1874 (two years before the organism *M. darwinii *was described as a new species), Haeckel concluded that the two-layered gastrula (i.e., the "Gastraea" or "Urdarmtier") is the ancestral form of all animals. This is the essence of Haeckel's Gastraea theory for the origin of the Metazoa (multicellular animals) [27], a concept that has been corroborated by numerous subsequent studies.

In Vol. 2 of his *Generelle Morphologie der Organismen *published in 1866, Haeckel outlined his "biogenetic law", which the author later described in more detail, in the following words: "Ontogenesis is the short and fast recapitulation of phylogenesis, controlled through the physiological functions of inheritance (reproduction) and adaptation (nutrition)" [28]. The significance of Haeckel's "law" (which is today down-sized to a "rule" that permits exceptions), with respect to the evolution of animals, has recently been described by Olsson et al. [29]. More importantly within the context of this article are the "genealogical trees" drawn and depicted by Haeckel in his classic monograph. In all of these tree-like diagrams, German oaks were used as the representative woody plant. This choice is not surprising. According to the pre-Darwinian idea of the *Scala Naturae *(Figure 2), the one primate of plants, organisms that were ranked in this Christian medieval hierarchy below the animals, were oak trees [10]. Since the British scientist Wallace likewise referred to the "twigs of a gnarled oak" [19], we have to conclude that so-called "ladder-thinking" was still alive in the minds of Haeckel and Wallace, who published major books on organismic evolution after the death of Darwin in 1882.

**Figure 6:**
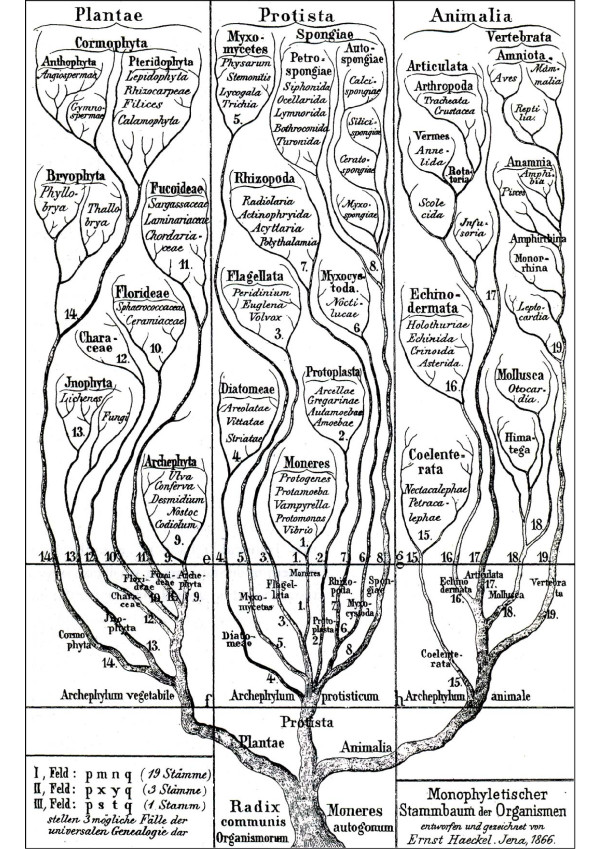
**Reproduction of Ernst Haeckel's genealogical oak tree depicting the Kingdoms Plantae (plants), Protista (micro-organisms) and Animalia (animals)**. Note that the author explicitly pointed out that this general Tree of Life is monophyletic [adapted from ref. 28].

The most prominent "general" evolutionary tree of Haeckel, depicting the presumed phylogenetic relationships between animals, plants and various "lower organisms" is reproduced in Figure 6. Three facts should be highlighted in this context. First, Haeckel [28] argued that the "Stammbaum der Organismen" (Tree of Life) is monophyletic. This hypothesis, which corresponds to Darwin's "species theory No. 2" (see Figure 4), has recently been corroborated by D. J. Theobald [30], based on protein sequence and other molecular data. The author concluded that "the last universal common ancestor [LUCA] may have comprised a population of organisms with different genotypes that lived in different places at different times" [30]. Second, in contrast to Darwin [6,7], whose work was based on the 19th-century "animal-plant-classification", Haeckel distinguished between three "Kingdoms of life":

1. Archephylum vegetabile (Plantae), 2. Archephylum protisticum (Protista), and 3. Archephylum animale (Animalia). Hence, unicellular "lower organisms" (Protista, inclusive of the Moneres, i.e., Bacteria), living beings that were largely ignored by Darwin [6,7], were present in Haeckel's view of biodiversity on Earth. Finally, Haeckel [28] coined the term "Moneres autogonum" to denote micro-organisms at the common root ("Radix communis Organismorum") of his "Tree of Life" (Figure 6). However, it should be noted that on other pages of his books, Haeckel [28] refers to polyphyletic origins of species. A discussion of all of Haeckel's pertinent ideas is beyond the scope of this article.

The inclusion of microbes that lack a true nucleus ("Moneres autogonum") into an evolutionary scheme was a large step towards our modern view of biodiversity. Today we know that the moneres (Kingdom Bacteria or Monera) are the dominant forms of life on Earth [31]. It is obvious that, via the inclusion of the Protista (i.e., micro-organisms with and without a nucleus), Haeckel [28,32] tremendously enlarged our view of life on Earth - the discipline of "Monerology" (i.e., Bacteriology and Protozoology) with respect to evolutionary questions rests to a large extent on the work of this famous German biologist. In some of his later writings, Haeckel mentioned the principle of endosymbiosis (or symbiogenesis), with reference to the origin of certain green algae. This topic is discussed in the next section.

## Constantin Mereschkowsky's symbiogenesis theory and the origin of eukaryotes

In a seminal paper published a century ago in German, the Russian biologist Constantin Mereschkowsky (1855-1921) wrote that the most important question of the biological sciences concerns the origin of species on Earth. However, according to Mereschkowsky [33], earlier attempts of Darwin and Haeckel were not successful, because "at the time when they were active not all the facts that are necessary to solve this problem were available. However, in the meantime novel facts from disciplines such as cytology, biochemistry, physiology, notably of the lower organisms, accumulated so that a new approach to solve the riddle concerning the origin of living beings is justified" [33].

As an alternative to the Darwinian principle of "descent with modification (i.e., biological evolution) by means of natural selection", Mereschkowsky proposed his theory of symbiogenesis [34,35]. This concept posits that new organisms, at the level of single cells, occur via symbiotic events, i.e., by means of the fusion and subsequent cooperation of microbes or "Moneren". Since the origin of the nucleus was one of Mereschkowsky's major topics, the term "symbiogenesis" includes "eukaryogenesis", i.e., the evolutionary development of nucleated cells from non-nucleated, bacteria-like ancestors. Hence, the original word "symbiogenesis" should be used instead of the more recently introduced term "eukaryogenesis" to denote those processes that led to the origin of the earliest nucleated (eukaryotic) cells [35].

Based on Mereschkowsky's insights and those of other cytologists, L. Margulis proposed the "serial endosymbiosis hypothesis of the origin of eukaryotic cells" that contain a nucleus and organelles (mitochondria, chloroplasts) within their cytoplasm [36,37]. The evidence for this version of the "symbiogenesis theory" has been summarized and discussed at length by Kutschera and Niklas [38,39], Cavelier-Smith [40-43], Koonin [44-47] an others [48,49]. As E. Koonin has recently stated in this journal, according to the well-supported "symbiogenesis sceniario", a single endosymbiotic event involving the uptake and subsequent domestication/enslavement of an alpha-proteobacterium by an archaebacterial host cell led to the generation of the mitochondria within heterotrophic eukaryotic cells. In a second step, the uptake of an ancient cyanobacterium, led to the origin of plastids (chloroplasts) [44]. These key events in the history of life on Earth (i.e., serial primary endosymbioses 1 and 2) occurred ca. 2200 to 1500 and ca. 1500 to 1200 million years ago, respectively, during the Palaeo- and Mesoproterozoic [38]. At that time, the oxygen content of the oceans was about to rise due to cyanobacterial photosynthesis. Gross and Bhattacharya [50] have proposed that the "birth of eukaryotes, a milestone in the evolution of life on our planet", was driven by the selective pressure caused by reactive oxygen species (ROS). These ROS were light-mediated by-products of the local rise in O_2_-levels within marine ecosystems during the Proterozoic.

**Figure 7:**
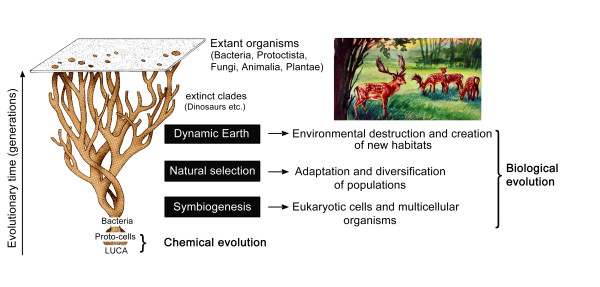
**Symbiogenesis, natural selection, and the dynamic Earth as key processes that caused biological evolution**. The Last Universal Common Ancestor (LUCA) evolved into the earliest self-replicating proto-cells (ancient microbes) ca. 4000 to 3500 million years ago. Over the subsequent eons, these archaic microbes evolved into numerous bacterial ecotypes that today inhabit every micro-niche where organic molecules (or light) are available. Moreover, these micro-organisms gave rise to larger, eukaryotic cells via symbiogenesis (primary endosymbiosis). These nucleated cells further evolved into multicellular organisms, such as algae, fungi, animals and plants.

Although many details concerning the evolutionary origin of the earliest nucleated, organelle-containing cells are still a matter of debate [51-53], there is agreement among scientists that symbiogenesis (primary endosymbiosis) was an early key process in the history of life [38,39,54]. However, although Mereschkowsky [33] was the first to clearly point out the importance of endosymbiotic events during evolution, he did not accept the "Darwin-Wallace principle of natural selection" [20] as a driving force for the transformation of species. This idea prevails to the present day: a number of "symbiogenesis-researchers" consider endosymbiotic events and directional natural selection as mutually exclusive concepts (see ref. 38 for a discussion of this topic). However, as documented in detail elsewhere [26,54,55], this view of the natural world is at odds with numerous observations and experiments. Symbiogenesis, i.e., primary (and secondary) endosymbioses, combined with directional natural selection caused by slowly changing environmental conditions, have been two key processes or "driving forces" of organismic evolution, since the origin of the hypothetical Last Universal Common Ancestor (LUCA) that gave rise to a heterogeneous population of aquatic, bacteria-like Proto-cells ca. 3800 million years ago [24,30]. These "factors" of biological evolution, with respect to the "Tree of Life", are depicted in Figure 7.

It should be noted that the relevance of endosymbiotic events, combined with the process of (bacterial) horizontal gene transfer, has already been discussed with reference to the "Tree of Life" [23-25,42,56]. However, a third key process shown in Figure 7, the movements of tectonic plates (i.e., the dynamic Earth), has been ignored by these investigators. The significance of these gradual (sometimes abrupt) changes in the environment with respect to biological evolution are discussed in the next section.

## Alfred Wegener's vision of the dynamic Earth, volcanism and plate tectonics

Four decades ago, J. C. Maxwell summarized the concept of the dynamic Earth in the following words: "The earth's surface, in the context of geologic time, may be likened to a boiling vat of maple syrup. The crust, with its high-standing continents, is analogous to the scum which rises from boiling syrup, coalesces, drifts apart, and rejoins in different patterns on the surface of the convecting liquid. The earth's crust is a similarly thin scum of relatively light rocks 'floating' on the mantle, a zone of heavier materials extending halfway to the earth's center and overlying the inner metallic core. By some cosmic accident the earth has been endowed with a magnetic field, apparently for much of its 4.5-billion-year history. Changes in the field with respect to a point on the surface are recorded by successively formed sequences of rock. Analysis of these ancient magnetic fields gives convincing evidence of extensive differential movements in the earth's crust. The composition of the crust and the forces which cause its deformation are apparently determined by gravitational and thermal instabilities within the outer few hundred kilometers of the mantle. Temperature within the earth increases downward at a rate exceeding the adiabatic gradient for the upper few hundred kilometers, hence this outer zone is intrinsically unstable. Vertical movements, once initiated, tend to be self-propagating. These instabilities may give rise to lateral and vertical movements approximating convecting currents in liquids. Rising currents have apparently occurred largely in oceanic areas, bringing new mantle material to the surface in the oceans and sweeping older oceanic rocks towards and perhaps beneath the high-standing continents. The great mountain ranges which border many continents are believed to be related in some way to the convective overturn of rocks in the earth's crust and upper mantle" [57]. This description, published in 1971, was a concise summary of a long series of articles and books that originated in 1858.

**Figure 8:**
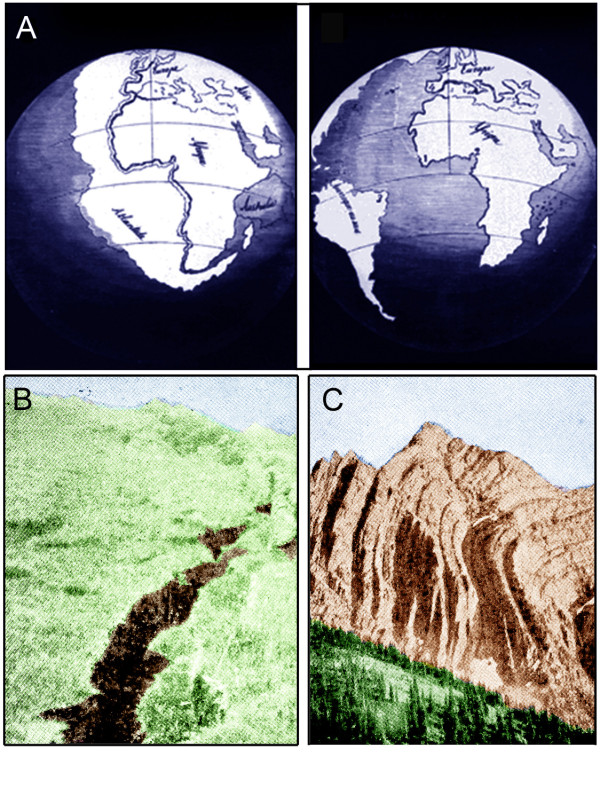
**Scheme depicting the idea of continental drift as envisioned by A. Snider-Pellegrini in 1858 **(A). This concept was re-discovered and supported by empirical evidence by A. Wegener in 1929. Decades later, the theory of plate tectonics was deduced. Plate tectonics accounts for most of the planet's earthquakes, which may result in deep cracks in the Earth's surface (B) and the formation of mountains as a result of horizontal compression of the crust (C) [adapted from ref. 58 and from photographs of the US Geological Survey, 1938].

In this year, the French geographer Antonio Snider-Pellegrini (1802-1885) published two imaginative maps depicting the continents of the Earth, "before and after separation" [58]. This early outline of the idea of continental drift, and hence the dynamic Earth (Figure 8 A), did not convince the geologists of the time, because Snider-Pellegrini's speculations were largely based on Biblical myths and only on few scattered empirical data. As a result, the old concept of a static Earth, the "geological basis" of all views of the "hierarchy and organization of life on our planet", as depicted in ladders and trees, from Charles Bonnet's *Scala *via Darwin's *Diagram *to Ernst Haeckel's *Oak*, prevailed (Figures 1 to 6).

**Figure 9:**
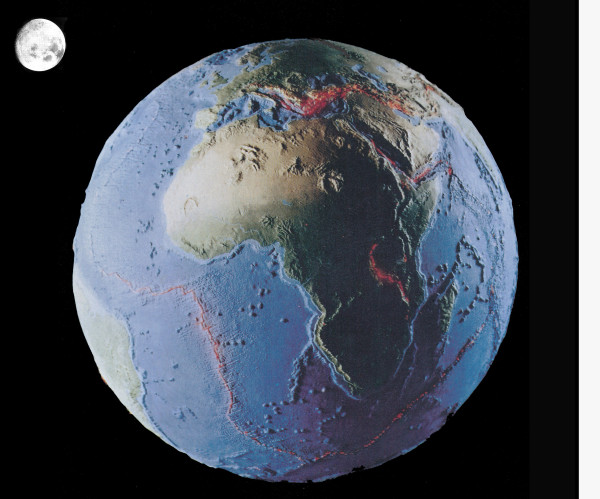
**Model of the Earth's surface, which is broken into drifting fragments, the so-called tectonic plates**. The "lubricant" of plate movements is liquid water. In this picture, the South American and African plates are highlighted. In addition, the moon, a solid satellite without water and plate movements, is shown in the upper left quarter. Red lines: Regions where volcanic eruptions occur frequently [adapted from ref. 75].

Seven decades after Snider-Pellegrini's account was published, the last (definitive) edition of Alfred Wegener's (1880-1930) book on *The Origin of the Continents and the Oceans *[59] appeared in print. In this monograph of 1929, the German scientist summarized a long list of empirical evidence for a novel fact-based theory of continental drift that overshadowed the earlier, Bible-inspired speculations of the French geographer. In essence, Wegener stated that the isolated continents as we observe them today were once united and formed a super-continent. This giant proto-land mass ("Pangaea") may have covered up to 50% of the surface of our planet and was surrounded by one large ocean ("Panthalassa"). Due to "continental drift" via mechanisms inexplicable to Wegener, the land masses finally reached, in the course of millions of years of steady motion, the position they have today. Despite Wegener's inability to explain the physical processes that may have caused the drift of these large land masses, the author proposed that the formation of mountains, via compressive forces, the occurrence of earthquakes, and volcanism are consequences of continental drift [59]. Today it is well established that Wegener was right (Figures 8 B, C; 9).

Only a few years before J. C. Maxwell published his summary of the "Dynamic Earth" quoted above, the concept of plate tectonics was proposed. This unifying theory of geology states that the Earth's outer rigid shell (i.e., the lithosphere) is broken into more than a dozen giant, rigid plates that float on the hot, ducile mantle (i.e., the asthenosphere) like pieces of ice on a lake. Most of the Earth's documented history results from plates rifting into pieces to form new ocean basins. When they converge back together, they can form mountains and large continents (Figure 8 C). As shown in Figure 9, the rigid lithospheric plates differ in size and their direction of internal heat-driven motion. Some pieces of the outer crust, such as the North American Plate, carry continents and attached pieces of the ocean floor. Other parts of the lithosphere, such as the Pacific Plate, are entirely covered by oceans and are made of oceanic crust. For instance, in the area of San Francisco (California) and elsewhere, the North American and Pacific Plates are pushed at each other, and the spontaneous release of pressure causes abrupt, short plate movements, so-called "earthquakes" (Figure 8 B). These rapid, unpredictable geologic events may have devastating secondary effects. For instance, the 2011 Sendai 9.0 megathrust earthquake that occurred on March 11 off the coast of Japan triggered destructive tsunami waves with highs of up to 12 m. These masses of sea water have travelled up to 10 km inland, destroyed the terrestrial landscape, and caused thousands of deaths. Frequently, such devastating earthquakes occur along the so-called "Pacific Ring of Fire", stretching from New Zealand, along the eastern edge of Asia north across the Aleutian Islands of Alaska and south along the coast of North and South America. The "Ring of Fire", which has 452 volcanoes, is a direct consequence of plate tectonics and hence the movements/collisions of crustal plates [60].

What is the significance of the theory of plate tectonics for the geological sciences? Theodosius Dobzhansky (1900-1975) once said that "Nothing in biology makes sense except in the light of evolution" [61]. Accordingly, earth scientists may conclude that "Not much in geology makes sense except in the light of plate tectonics". In other words, the theory of plate tectonics is the unifying principle of historical geology.

The consequences of the internal heat-driven movements of tectonic plates for the evolution of life on Earth, as well as the "Tree-models" depicting this process, are obvious: new habitats are created and existing ones are re-modelled or destroyed by these geologic events (Figure 7). Major mass extinctions, such as those that occurred 251 and 65 million years ago, respectively, were at least in part caused by massive volcanic eruptions and hence the dynamic Earth [54,60].

**Figure 10:**
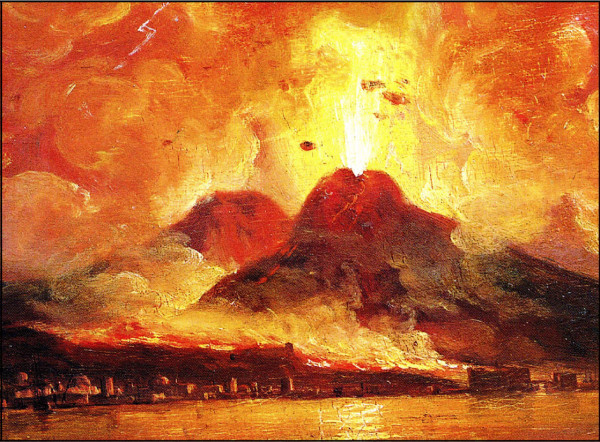
**The massive 1872 eruption of the Vesuvius, the only active volcanoe in mainland Europe (Italy)**. Vesuvius is most famous for the 79 A. D. eruption that destroyed the Roman cities of Pompeii and Herculaneum. Plate tectonics is the major cause for these violent eruptions, which document ongoing magmatic processes driven by heat from the radioactive decay within the Earth [adapted from an anonymous painting, ca. 1880].

**Figure 11:**
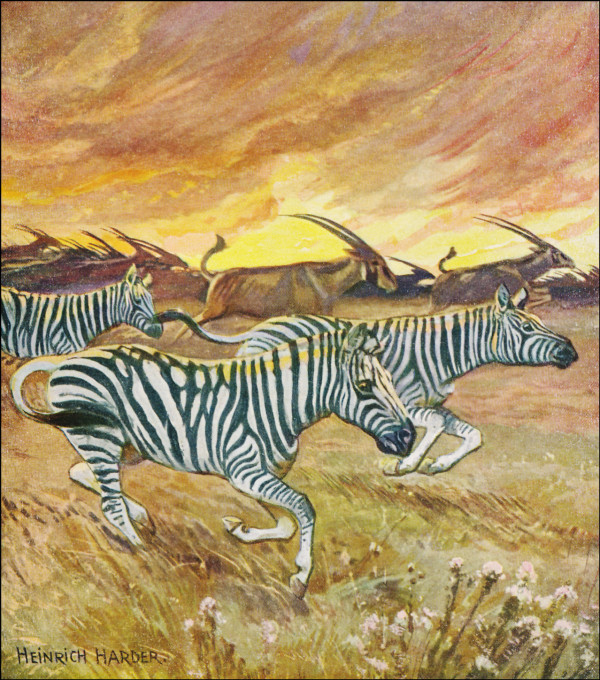
**Volcanic eruptions can ignite wild fires that result in the destruction of the vegetation, soil micro-organisms, and less mobile animals**. In this drawing, Zebras and other mammals are depicted that are just about to escape from a severe wild fire [adapted from a drawing of H. Harder, 1912].

In a recent publication it was documented that the break-up of the super-continent Pangaea (which existed from the Perminan into the Jurassic, ca. 299 to 200 million years ago), due to plate tectonics, accounts for the evolutionary diversification of many groups of animal, such as dinosaurs, mammals and the land leeches of Madagascar [62]. Another example for the role of plate tectonics as driving force for speciation are amphibious tetrapods, such as salamanders. A detailed analysis of numerous collected specimens of the four-toed Asian salamanders (family Hynobiidae) revealed that the 46 biospecies were "created" as a result of plate tectonics. About 110 million years ago, much of Asia was a low-lying humid region where salamanders of all varieties existed. A series of geologic events, which resulted in the lifting up of the Tibetan Plateau and mountain-building led to the isolation of sub-populations that evolved, over millions of years, into separate, geographically isolated species [63]. Hence, the dynamic Earth must be interpreted as a major factor that drove the evolutionary diversification of many macro-organisms on our planet [64,65] (Figures 8, 9). It should be mentioned that wildfires, which have a large impact on the distribution and diversification of plants and animals, are regularly caused by massive volcanic eruptions (Figures 10, 11). These secondary consequences of plate tectonics may also have been an important cause for the extinction of the dinosaurs that occurred 65 million years ago [54,61,66].

Finally, we have to address the question as to the consequences of volcanism (and the associated wildfires) on the evolutionary patterns of micro-organisms, such as bacteria, soil amoebae and unicellular algae (diatoms etc.) [67]. One case study may illustrate this topic. The eruption of Mount St. Helens in southwest Washington, USA, on May 18, 1980 released superheated steam and gases. Moreover, this catastrophic event resulted in pyroclastic flows, landslides, mudflows, and ash fall. As a result, novel habitats were formed, whereas old ones were re-structured, scoured, or eliminated [68]. Six years after the eruption, some aquatic habitats were analyzed with respect to the presence of micro-organisms. The results show that species richness and microbial diversity were very low at the most heavily disturbed sites around the cool volcano, documenting a large mass extinction event at the "micro-scale" [69]. However, more work on other volcanic sites is necessary to corroborate these results. These data document that volcanic eruptions (and wildfires) lead to a temporary "sterilisation" of the affected aquatic and terrestrial habitats and hence to the destruction of most of the micro-organisms that existed there before the catastrophic event occurred (Figures 10, 11). The patterns of re-colonization by microbial communities and the resulting evolutionary diversifications are not yet explored in detail.

## Conclusions: The tree-like Synade-model of macroevolution

In a recent analysis of Charles Darwin's "species book" it was documented in detail that the British naturalist and theologian used Biblical phrases such as "He who ..." throughout his *Origin of Species *[70]. Darwin's key metaphor for the principle of descent with modification, combined with his theory of the last common ancestor, was the "great Tree of Life" [6,7]. In this context I would like to add that the symbol of Trees appears in the creation myth of the Old Testament (Genesis 2, 9): "And the Lord God made all kinds of trees grow out of the ground - trees that were pleasing to the eye and good for food. In the middle of the garden were the tree of life and the tree of the knowledge of good and evil".

In this article I have shown that, from the earliest, Bible-inspired "Moral Tree" (Figure 1), via the hierarchical *Scala Naturae*, to Darwin's and Haeckel's static trees (or corals) of life, the old, Biblical "woody plant-model" evolved by descent with modification: The Christian "ladder-thinking" was gradually replaced by the post-Darwinian ("Haeckelian") atheistic "oak-tree-concept" that included animals, plants and micro-organisms (Figure 6). However, neither Darwin nor Haeckel took the principle of symbiogenesis (primary endosymbiosis) into account, because this evolutionary process - the "creation" of more complex eukaryotic cells via the fusion of archaic microbes and the subsequent cooperation of the partners - was largely unknown at that time. It should be stressed that Haeckel mentioned symbiogenetic events in the context of the origin of green algae and land plants [34], but the German biologist failed to integrate this insight into his general picture of the evolution of life on Earth.

Moreover, the trees of Darwin and Haeckel are "static", based on their implicit assumption of an Earth surface that does not display significant movements. Unfortunately, even the "architects" of the Synthetic Theory of Biological Evolution developed between 1937 and 1950, Theodosius Dobzhansky (1900-1975), Ernst Mayr (1904-2005), Julian Huxley (1887-1975), George G. Simpson (1902-1984), Bernhard Rensch (1900-1990), and G. Ledyard Stebbins (1906-2000) ignored symbiogenetic events and the dynamic Earth [54,61,71-73]. These fundamental processes were, like the insights gained from the disciplines of evolutionary developmental biology ("Evo-Devo") and geology (mass extinctions), integrated into the "Expanded Synthesis" published in 2004 [54,74]. Due to this steady growth of our "Tree of modern evolutionary knowledge", the scientific discipline of evolutionary biology has been defined as a "system of theories" that explains the various aspects of those processes that Charles Darwin described as "descent with slight and successive modifications" [6,7].

W. F. Doolittle [23-25,56] and others [52] have recently argued that the construction of a universal Tree of Life, as originally suggested by Darwin [6,7] and Haeckel [28] (Figures 4 and 6), may be difficult to achieve. These authors based their judgement on two facts. First, in prokaryotes (bacteria, cyanobacteria), which comprise the majority of life forms on Earth and were the sole organisms during ca. 2/3 of the early history of organismic evolution on this planet, lateral gene transfer (the exchange of genetic information between extant microbes) occurs regularly. Second, endosymbiotic events, and hence the fusion of microbial lineages, should be taken into account when tree-like models are drawn [51].

In my view, symbiogenesis, denoted here as early primary endosymbiotic processes that gave rise to the organelle-bearing eukaryotic cells during the Proterozoic, and subsequent, secondary endosymbiotic events that are responsible for the origin of the majority of the unicellular marine phytoplanktonic organisms [38,39,54,74], were key events during the history of life. Moreover, plate tectonics and hence the dynamic Earth must be incorporated into our view of any tree-like reconstruction of biological evolution. Based on the facts summarized in this article and elsewhere [5,62,75] I propose that symbiogenesis, (directional) natural selection, and the dynamic Earth were key processes that must be viewed as three important "driving forces" of organismic evolution (Figure 7).

**Figure 12:**
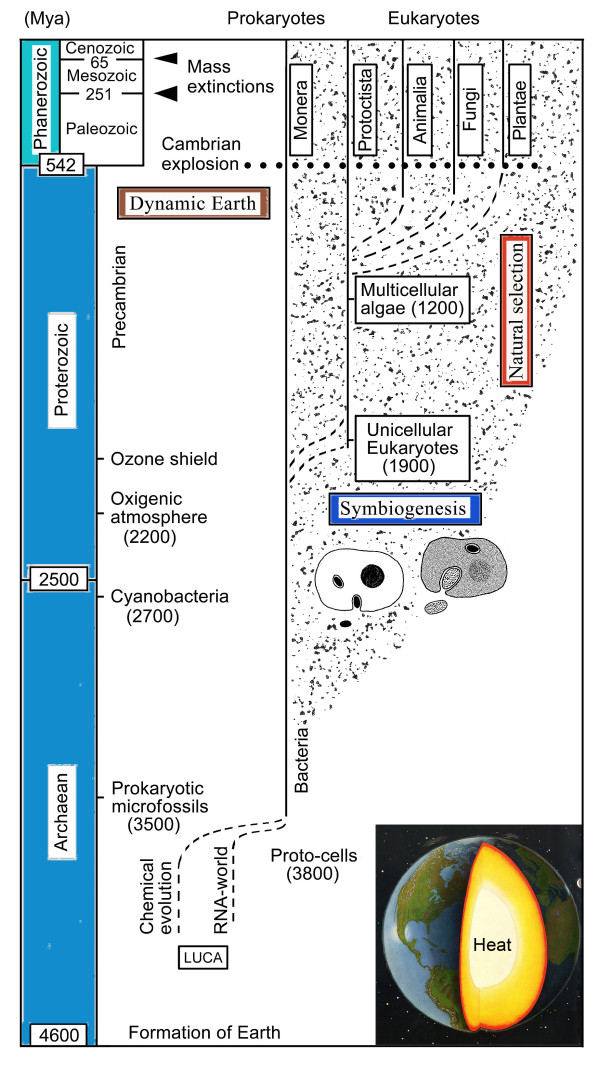
**The tree-like "Synade-model of macroevolution", taking into account all five Kingdoms of life on Earth**. According to this theory, symbiogenesis (primary and secondary endosymbiotic events), (directional) natural selection, and the (internal heat-driven) dynamic Earth were and still are key drivers of macroevolution on our "planet of the bacteria" [adapted and modified from ref. 62].

A more precise "tree-like" version of this "synade-model" of macroevolution, which takes into account all organisms on Earth (i.e., members of the Kingdoms Bacteria, Protoctista, Animalia, Fungi, and Plantae) is depicted in Figure 12. The oldest branch of living beings, the Bacteria (syn. Kingdom Monera), represent more than 50% of the protoplasmic biomass on Earth [31]. They are, as pathogens and/or symbionts, important "factors" in the evolution of all Eukaryotes. Hence, ancient and recent prokaryotic microbes are included as "background organisms" [31,67]. In addition, a scheme of our planet depicting the centre of the Earth is shown in Figure 12. Without internal heat, which is primarily caused by the energy given off as a result of the radioactive decay of uranium, our "blue planet" would probably be as static as the moon (Figure 9).

According to the "tree-like" synade-model of macroevolution proposed here (Figures 7 and 12), all extant and extinct organisms have, through the eons of geological time, benefited from the dynamic Earth due to the creation of early terrestrial land masses within giant marine habitats, and the subsequent formation of mountains, deserts, freshwater ecosystems, and deep oceans [64,75]. On the other hand, massive volcanic eruptions, which are "side effects" of plate tectonics, have caused (or significantly contributed to) several mass extinctions during Earth's history. Hence, the mobile tectonic plates led to the destruction of countless living beings on this "blue planet of the microbes". Finally, it should be stressed that micro-organisms, the "unseen majority" [31] that Darwin largely ignored [76,77], are the true "winners" in the ongoing, ca. 3.500 million-year-long "struggle for life" on our ever changing, dynamic Earth [5,59-62,78-86].

## Reviewer's comments

### Reviewer 1: Mark Ragan, University of Queensland, Australia

*Reviewer's comments: *In this article the Author develops a new perspective on macroevolution, based on a narrative of how our understanding of the living world has evolved particularly over recent centuries. The main models along this trajectory are a static Chain of Being, a static Tree of Life, and finally a geologically (hence environmentally) dynamic tree-like "synade" driven primarily by symbiogenesis, "directional" natural selection and plate tectonics. I completely agree that it is important to situate our understanding, not least about evolution, in historical perspective, and the Author rightly attempts to weave together this history from developments in two fields, geology and biology. He further reminds us of the broader contexts of the intellectual enterprise, referencing philosophy, religion, human behaviour as well as scientific content *per se*. Addressing this important challenge requires broad but careful interdisciplinary scholarship, and my comments largely focus on issues of historical accuracy, not on the (undisputed) merit of the undertaking itself.

The author claims that the *Scala Naturae *was a "Western medieval Christian" idea. Lovejoy (*Great Chain of Being*, 1936) and others have clearly shown that ideas of perfection, vertical ordering and continuity long preceded Christianity. The Chain of Being infuses the teachings of Pythagoras (Sixth century BCE), and Paul Kuntz (*Jacob's Ladder*, 1987) finds it common to most religious traditions. In any event there is substantial overlap between the various canons of the (Christian) *Old Testament *and the (Hebrew) *Tanakh*. Nor is it helpful to refer to the Chain of Being as Christian "dogma" (a belief that cannot be doubted, against a penalty of excommunication).

*Author's response: *I agree with the opinion of the referee and have modified the text accordingly.

*Reviewer's comments: *Regarding Lull (or Llull), it is a little imprecise to say he *published *a tree-like scheme, as the printing-press came into use more than 120 years after his death. Trees of logic, *divisio scientiarum *and genealogy appeared many centuries before Lull. Nor was Lull's diagram unique in admixing the real with the supernatural, mystical or conceptual (*cf*. Jacob's Ladder, medieval cosmologies, sephirotic trees). The Author correctly states that Lull had only limited influence, but goes on in the same sentence to claim that nonetheless "...the Western medieval Christian idea of the *Scala naturae*... is unequivocally related to the Bible-based ideology depicted in Figure 1" [Lull's apostolic tree]. Precisely what does "related to" mean here?

*Author's response: *The text was re-written in order to clarify these points.

*Reviewer's comments: *The Author incorrectly characterises Bonnet as having believed that "there is no change in nature, everything remains identical, and species are constant". Bonnet did indeed embrace a static framework of increasingly perfect steps that we recognise as biological taxa, but wrote (*Palingénésie philosophique*, 1769) that the germ of each form of life gradually progresses within this framework, *i.e*. works its way step-by-step up the Chain. Buffon had entertained similar ideas slightly earlier, as would Lamarck later. The Author's Figure 2 shows only the top half of Bonnet's chain of being, which was first published in 1745 (*Traité de Insectologie*), and later (1779, not 1719) appeared in the first volume of his *Oeuvres*.

*Author's response: *The text was modified as recommended and the legend to Figure 2 corrected.

*Reviewer's comments: *I do not understand how any branched tree can be said to be "more ladder-like than a true tree with branches", nor how Lamarck's 1809 and 1815 trees are topologically any more ladder-like than the one in Darwin's *Origin*. Trees have branches, ladders do not, and Lamarck's diagrams show branches.

*Author's response: *These unclear sentences were re-written and ... "branches with twigs" added so that this misunderstanding is now corrected. In my view, Lamarck's table of 1809, reproduced in Figure 2, looks more like a ladder than a "true tree". For instance,/Infusoires, Polypes and Radiaires/,/Insectes, Arachnides, Crustacés/and/Annelides, Cirrhipèdes, Mollusques/are depicted as ladders, but no such A/B/C-arrangement can be found in Darwin's tree reproduced in Figure 4. However, I agree with the referee that this is my own personal view and one can likewise imagine a tree in this static Tableau.

*Reviewer's comments: *The Author refers to Cuvier's "religious" opposition to Lamarck. While Cuvier was a Protestant and Lamarck, born into a Catholic family, became a deist, their famous antagonism was above all else a "competition for priority and empire" (Outram, *Georges Cuvier*, 1984). To my (admittedly limited) knowledge, Cuvier was not similarly belligerent toward other non-Protestants; nor am I aware that he criticised Lamarck for his religious beliefs *per se*.

*Author's response: *This sentence was modified as follows: After his death, Lamarck encountered fierce religious and political opposition.

*Reviewer's comments: *The Author insists that Darwin's use of "old-fashioned terms such as 'perfection, improvement, higher *vs *lower (or primitive) forms of life' ... clearly documents relicts of religiously motivated 'ladder-thinking' in his mind". A scan of the *Origin *for occurrences of *perfect *finds that Darwin used it to describe structural complexity, specialisation and competitiveness in the struggle for existence - arguably fair use for a general audience in 1859, and utterly distinct from its sense in Bonnet, much less Bonaventure. Nor were many specialist terms available - recall Haeckel's orgy of neologism in *Generelle Morphologie *(1866). While I don't deny that Darwin, like us all, was a product of his intellectual place and time, the Author fails to make his case that these terms arose from ladder-thinking *per se *and not simply from the broader Western intellectual tradition (*e.g*. the idea of perfection in *Timaeus*).

*Author's response: *I agree that my conclusion is not supported by strong evidence and have therefore modified the text accordingly.

*Reviewer's comments: *The Author takes Darwin to task for failing to mention bacteria in his published work. Microscopic life had long been imagined (Ovid, Pliny), and its eventual observation (Leeuwenhoek, mid-1670s) prompted Thomas Hobbes to praise the majesty of God revealed in His microscopic creation (*Decameron Physiologicum*, 1678). By 1859 microbial life was known to be abundant and diverse, although bacteria *per se *remained poorly known: Pasteur began his work on bacteria in the early 1860s, Koch from the mid-1870s, and Cohn's classifications date from 1872. O'Malley (*Trends in Microbiology *17:341-347, 2009) documents that Darwin was well aware of progress in microbiology, corresponded with Ehrenberg and Haeckel, and even invited Cohn to Down House. Whether because he concluded that the state of knowledge would not advance his argument, or judged the area as too unfamiliar to his audience, Darwin omitted mention of microbes in the first edition of *Origin*. Responding to criticism, however, he added text on "some of the lowest forms, as the infusoria and rhizopods" for the third edition (1861).

*Author's response: *I have modified the text and suggested that Darwin's remarks on microbes are confusing and unclear.

*Reviewer's comments: *The Author rightly calls attention to Haeckel's acknowledgement of Lamarck: the second volume of *Generelle Morphologie *(1866) was dedicated to Darwin, Lamarck and Goethe. Readers with German may be interested in Haeckel's *Die Naturanschauung von Darwin, Goethe und Lamarck *(1882).

*Author's response: *In Germany, most of the books of Haeckel, which were sometimes distributed via local publishers, are no longer available. Moreover, no complete list of publications (papers, books, monographs) has ever been published, due to a lack of interest in this eminent biologist in this country - a "Haeckel-Industry" does not exist in Germany. Unfortunately, I am unable to purchase a copy of Haeckel (1882) and study this "Schrift".

*Reviewer's comments: *The Author refers to the oak tree as "the primate of plants" in the "Christian medieval hierarchy" of the *Scala Naturae*, and on this basis reads Haeckel's choice of the oak for his classic tree-diagrams, and an 1855 comment by Wallace, as examples of atavistic "ladder-thinking". I know of very little evidence that the oak tree was widely considered as the most-perfect plant, hence to link vegetables with animals. Quite the opposite: for Bradley (*Philosophical Account*, 1721) the most-perfect plant was the fig; for Linnaeus (*Ordines Naturales*, 1764) the palms; for Bonnet (*Contemplation*, 1764) *Mimosa*; for Oken (*Elements*, 1847) the apple; for Haeckel (*Natürliche Schöpfungsgeschichte*, 1868) the bell-blossom; for A.P. de Candolle, Endlicher and E. Fries the Ranunculaceae, Papilionaceae and Compositae respectively, with Nägeli (*Mechanisch-physiologische Theorie*, 1884) tending to support Fries. Cotton Mather (*Christian Philosopher*, 1721) lauded the cabbage-tree and banana, while in a paragraph beginning "Even the most *noxious *and the most *Abject *of the *Vegetables*..." described fungi in "rotten Barks of Oaks".

*Author's response: *In Germany, Oaks are the symbol of strength and beauty. I believe that, for this reason, the German biologist Haeckel used the Oak in all of his drawings wherein he depicted phylogenetic trees.

*Reviewer's comments: *The Author claims that Haeckel argued that the Tree of Life is monophyletic, and to be sure, trees depicting a monophyletic origin of organisms appear in his work (*e.g. Natürliche Schöpfungsgeschichte*, opposite page 569). In two other places even in this same book, however (pages 347, 382), Haeckel depicts multiple origins *via *spontaneous generation, with nine and six lineages respectively persisting to the present. His discussions in *Protistenreich *(page 66) and especially *Generelle Morphologie *(Vol. I, chapter 7) are nuanced, but he comes down on the side of polyphyly. By the sixth edition of *History of Creation *(Lankester's translation of the eighth edition of *Natürliche Schöpfungsgeschichte*), Haeckel finds the question to be "of very subordinate importance" (Vol. II, page 42).

Theobald's argument is powerful, but few things in natural history are "unequivocal".

*Author's response: *I have added the sentence "However, ..." and extended the legend to Figure 6 accordingly. The statement concerning ref. 30 was modified.

*Reviewer's comments: *The Author correctly states that Haeckel included both nucleate and anucleate micro-organisms in his Protista, but most subsequent authors restrict the term to morphologically simple eukaryotes. To my knowledge Haeckel never used the term *Monerologie *and, given potential confusion with *Monadologie *(Leibniz) or *Monismus *(Haeckel again), I caution against its adoption.

*Author's response: *I think that the term "Monerology" is justified, because, as a result of Haeckel's work, the study of the Monera gathered momentum. Therefore, the text was not modified as recommended by the referee.

*Reviewer's comments: *The idea that organisms were or continue to be assembled from free-living units can be traced to Schimper (1883, 1885) and Oken (*Die Zeugung*, 1805), indeed to various seventeenth-century corpuscular theories. As the Author states, Mereschkowsky put symbiosis centre-stage as the key to cellular origin, and a unifying evolutionary principle. It is, however, imprecise to say that Mereschkowsky's *symbiogenesis *includes eukaryogenesis, as the term *Eucaryotes *was introduced by Chatton only in 1925. The 1992 translation of Khakhina's *Concepts of Symbiogenesis *(Russian original 1979) is an indispensable reference on Mereschkowsky and his period.

*Author's response: *I disagree with this opinion. In Mereschkowsky's paper the origin of the nucleus is discussed at length. Hence, symbiogenesis includes eukaryogenesis, as described in the text. The book of Khakhina (1992) is cited and discussed in detail in ref. 35 (in German).

*Reviewer's comments: *I disagree with the Author that a "static Earth" prevailed in geology pre-1858. In fact the previous decades had been characterised by intense debate: uniformitarians (Lyell, Chambers) *versus *catastrophists (Cuvier, Sedgwick, Whewell), Neptunists (Werner, Jameson) *versus *Vulcanists (Hutton, Playfair), Deluge universalists (Buckland) *versus *regionalists (Hugh Miller). Many, perhaps all, of these competing explanations were presented by their respective proponents as consistent with the Biblical account.

*Author's response: *According to Wegener (1929), ref. 59, the "static earth-view" was the dominant hypothesis of most scientists before ca. 1858. However, I agree with the referee that some geologists may have published "dynamic Earth-like" hypotheses and ideas before 1858, that are not cited anymore. The principle of natural selection was likewise proposed before Darwin (1859) by many authors. A detailed description of all of these concepts is beyond the scope of this article.

*Reviewer's comments: *I'm unfamiliar with this earth-science variant of Dobzhansky's saying (which, by the way, is slightly misquoted), and ask the Author to provide a citation. I don't dispute the importance of plate tectonics, but does it entirely crowd out, say, Steno's Laws as the sole "unifying principle of historical geology"?

*Author's response: *I have re-written this sentence accordingly.

*Reviewer's comments: *Figures 8, 9, 10, 11 are simply illustration, not scientific argument.

*Author's response: *In view of the devastating 2011 Sendai megathrust 9.0-earthquake and tsunami that occurred on March 11 in Japan (briefly discussed in the revised text) these figures are important illustrations of the most significant arguments of the paper. The text was extended with reference to the catastrophe that occurred in Japan.

*Reviewer's comments: *The Author cites himself (reference [70]) as documenting that Darwin (now additionally identified as a theologian) "used Biblical phrases such as 'He who...' throughout his *Origin"*. This raises the not insignificant matter of what constitutes a Biblical phrase, or more precisely a Biblical turn of expression. Darwin was familiar with the King James version (KJV, 1604-1611), while the phrase "He who laughs last, laughs longest" was in use by 1608, implying that "He who..." phrases entered the KJV from the broader language, not *vice-versa*. Many of course originated as Latin as "Qui..." phrases, for example "He who dares, wins" (*Qui audet, vincet*). "Qui..." expressions are not uncommon in the works of (or attributed to) *e.g*. Publius Syrus, Ovid and Seneca, and thus predate the Vulgate by 450-600 years. Darwin is likely to have encountered these in his schooling (in his *Autobiography *he refers, somewhat disparagingly, to his classics training at Cambridge).

*Author's response: *I agree with the referee: Darwin may have been indoctrinated with these Biblical "He who"-phrases during his education. However, it is clear that these "He who"-phrases can also be found in non-Biblical texts such as those cited above. Since Darwin was a theologian by training I have restricted my discussion to Biblical phrases.

*Reviewer's comments: *In the same passage, the Author adds that "the symbol of Trees first appears in the creation myth of the Old Testament". I remind the Author that Enkidu and Gilgamesh (27th Century BCE) felled the giant cedar to serve as the door to the Temple of Enlil - a symbolic use if ever there were one. A sacred tree was associated with King Ashurnasirpal II of Assyria (883-859 BCE). Moses is considered to have lived around the 14th Century BCE, and the books of the Pentateuch/Torah were written quite a few centuries later. For early tree-symbols in other cultures and contexts see Cook, *The Tree of Life: Image for the Cosmos *(1974).

*Author's response: *I agree with the referee and have removed the word "first".

*Reviewer's comments: *The Author asserts that "the trees of Darwin and Haeckel are 'static', based on their implicit assumption of an Earth surface that does not display significant movements." Haeckel in fact corresponded with Philip Sclater (Secretary of the Zoological Society of London 1860-1902, and founder of *The Ibis*) concerning a supposed lost continent (*Lemuria*) that had once served as a land bridge between Madagascar and India (explaining the modern distribution of lemurs) but was supposed to have since subsided. Nor is it necessary to invoke plate tectonics to bring dynamism into species trees: dispersal and allopatric speciation provide that, at least for macroscopic organisms.

*Author's response: *I have read the books of Darwin and Haeckel very carefully: In no section or sentence a dynamic Earth is mentioned, i.e., these biologists assumed that the earth is static (see Wegener 1929, who documents, in the first sections of his book, that, in the 19 th century, the "static Earth-view" prevailed). However, I agree with the referee that, in their notebooks etc., both Darwin and Haeckel may have envisioned a dynamic Earth. To the best of my knowledge, there is no proof for this speculation.

*Reviewer's comments: *The Author concludes by proposing that symbiogenesis, "directional" natural selection *in sensu *Weismann and Schmalhausen, and the dynamic Earth "must be viewed as the three most important 'driving forces' of organismic evolution." I admit to scepticism about Top Three lists, but if we're to play the game, then why, after (wrongly) excoriating Darwin for ignoring microbes, and calling Earth our "planet of the bacteria", does the Author select such eukaryote-centric processes? What about genetic variation, barriers to gene flow, energy flow through ecosystems, or planetary climate change? Or might we embrace Top Three List pluralism, according prokaryotes a separate set of drivers that might include surface-volume ratios, extrachromosomal inheritance and/or lateral genetic transfer?

*Author's response: *I fully agree with the referee and therefore have removed "the most" so that this sentence is now less "dogmatic". We are eukaryotic macro-organisms, with domesticated bacteria (mitochondria) in all of our body cells. Therefore, an "eukaryote-centric" view may be justified.

### Reviewer 2: W. Ford Doolittle, Dalhousie University, Halifax, Canada

*Reviewer's comments: *I very much enjoyed this thought-provoking essay, as, I am sure, will other readers of this issue of Biology Direct. So let's get right to some of the thoughts it provokes.

*On causes of evolution*. In general it is hard to complain about the general notion here, which is that the neo-neoDarwinian synthesis for this post-postgenomic era will be further loosened by the admission that symbiogenesis, reticulation at the gene level and a sort of born-again catastrophism will be (in fact long have been) muscling their way on to the center of the explanatory stage as causes of evolution. It is possible to complain, though, that Kutschera and so many others who promote this or that process as the or (even just one of the) principal causes of this or that pattern in evolution seem to believe that the idea of cause, and in particular the notion that we might rank causes by relative importance, is unproblematic. A search for "causation" in the online Stanford Encylopedia of Philosophy reveals that it is not, and especially when there are no conceivable common units of measure with which the complex of factors behind everything that has happened in the history of life on this earth might be quantitatively compared. Symbiogenesis, if seen as a natural predisposition or drive on the part of organisms is obviously out, but as a frequent solution to evolutionary challenges may well need more emphasis. The impact of spectacular but localized geological events on microbial evolution has certainly not been thought much about, maybe because of the belief that everything is and always has been everywhere.

*Ladder-of-life-ism*. I'd agree with the Dr. Kutschera that Darwin had not given up the notion that evolution is in some ways progressive, and it is common, as the author does, to deride such residual ladder-of-life or great-chain-of-being type of thinking. But we do all imagine that when life began it was very much simpler than today. Many contemporary biologists accept the ideas developed by Eors Szathmary and the late John Maynard Smith in their book *The Major Transitions in Evolution*. Indeed symbiogenesis itself of one of the examples of contingent irreversibility Szathmary and Maynard Smith stress, as a rung on what looks very like a ladder of advancing complexity. The Great Chain of Being, like Ontogeny Recapitulates Phylogeny, is something we are not supposed to believe in but that points obliquely to a truth. Maybe, as Dr. Kutschera claims, the old, bad, ladder-of-life thinking that he feels still infected Darwin was religious in character. But a new understanding of the inevitable increase in complexity is not, surely.

*LUCA*. Another point I and other enthusiastic lateralists might disagree with this author is in his claim that Douglas Theobald, in his recent Nature paper, confirmed Darwin's "species theory N. 2" ("the principle of the last common ancestor of all forms of life"). We'd agree that this was Darwin's idea, but not that it is true or that Theobald confirmed it. In fact, he (Theobald) writes that "the last universal common ancestor [LUCA] may have comprised a population of organisms with different genotypes that lived in different places at different times." This doesn't fit any reasonable definition of ancestor and in fact the model for evolution that several of us hold boils down to saying that LUCA is still alive and well. It is necessary to realize that having common *ancestry *does not logically entail sharing a common *ancestor*.

*Author's response: *I have re-written the unclear sentences accordingly and cited the important book of E. Szathmary and I. Maynard Smith (1998). The key sentence of the paper of Theobald (2010) was included.

### Reviewer 3: Staffan Müller-Wille, University of Exeter, United Kingdom

*Reviewer's comments: *This article presents some interesting suggestions as to the chief ingredients of contemporary attempts to tell the history of life on earth - natural selection, symbiogenesis and plate tectonics - but it fails miserably as a piece of intellectual history. I do not have the expertise to judge the "synade model" of macroevolution Kutschera proposes, but as a historian of the life-sciences I would like to point out some of the many inaccuracies and weaknesses of argument that permeate his account of how, in his own words, "the old Biblical 'woody plant-model' evolved by descent with modification." I will conclude with a few words on why I think it is important for contemporary biologists and their audiences to get the history of their discipline right.

I will begin with one of those details that historians like to fuss about. Figure 1 of Kutschera's article shows, as the caption describes it, "the 'Tree of Knowledge', drawn ca. 1304 by Raymon Lull (1235-1315)" (the correct, Catalan spelling of his name is Ramon Llull). Any attentive beholder will notice immediately that the "drawing" is in fact a woodcut, a technique Europeans began to employ around 1400 only, that is, almost a century after Llull had died. The image is, of course, taken from a posthumous print edition of Ramon Llull's *Arbor Scientie *that appeared in Lyon in 1505. And it does not represent the tree of knowledge but - as a heading in the original book indicates - a "moral tree". Latin inscriptions in the original, erased from the reproduction Kutschera uses, indicate that the fruits on the right-hand side of the tree - i.e., the tree's actual left-hand side - symbolize the seven deadly sins (wrath, greed, sloth, pride, lust, envy, and gluttony) growing out of corruption (*vitiositas*) which in turn roots in malice, stupidity, falsehood and absence of (good) purpose (*privatio finis*). Correspondingly, on the left-hand side of the tree we find the seven cardinal virtues, held securely in the ground by many more roots, including kindness, magnanimity and wisdom. The figure on top of the tree is of course not God, but Jesus, pruning the tree on its 'evil side' with the help of an axe (Figure 1).

The example demonstrates that Ramon Llull was able to exploit visual tree-metaphors in a number of ways, and that it is worthwhile attending closely to details and context [87]. In the case of the moral tree, the tree is actually *split *into two, and the basic idea illustrated by the tree metaphor is a *dynamic *one: virtue breeds virtue, and vice breeds vice, so that Jesus has to return to his worldly father's profession. Llull is also known for representations of the scale of nature (Figure 2), but in this case - unlike his "moral tree" - the underlying logic has very little to do with "Bible-based ideology", as Kutschera claims. The idea of a scale of nature *pre-dates *Christianity, with Plato as its first systematic proponent [88]. It builds on a simple intuitive idea, namely that diversity derives from the presence or absence of qualities and capacities, from "privation" and "perfection" in contemporary parlance. Take away rationality from a human, and you end up with an animal, take away sensation and locomotion, and you end up with plants. This has a lot do with ideology, but also with basic logic, yet very little specifically with the Bible or the Christian creed. Note that God, or Jesus for that matter, is conspicuously absent from Llull's scale of nature. The "highest" position is taken in by "Being (Ens)", i.e. substance in its most general sense, the "lowest" by individual humans like Plato and Socrates. If one prefers to read the "tree" top-down, an entirely materialist picture presents itself, with two pagan philosophers occupying the highest place.

Still, Christianity seems to be the main-culprit in the rather linear and static history that Kutschera himself tells about the evolution of tree-of-life-ideas. To call Llull a "fanatic Christian" is ludicrous. His doctrines were in fact condemned by papal decree in 1376. Georges Cuvier's opposition to Lamarck did certainly not lead to a return to "Biblical myths." Quite on the contrary, the morphological evidence he had gathered for the existence of four "embranchements" in the animal kingdom - vertebrates, molluscs, articulates, and radiates -, dealt the final death-blow to the idea of a scale of nature. When Darwin criticized "independent species creations" he did not have in mind "religious dogma" in general, but the theory that his friend Charles Lyell had laid out in his three-volume *Principles of Geology *(1831-1833). The diagram in Darwin's *Origin of Species *will only appear as a straight-forward tree, if one cuts it down like Kutschera does in Figure 4 - the original illustration shows a structure with no trunk but multiple roots (Figure 3). Darwin did include microbes in his scientific work [77], p. 229, and his view of our planet was certainly not static. And finally, while he did speak in terms of perfection and improvement, he handled these terms relationally, and shared with his contemporaries and predecessors, at least since Carl Linnaeus (1735-1778), a curious predilection for levelling out the presumed steps in the scale of nature, for example by demonstrating that earthworms possess intelligence in his last book [89], p. 189. In an almost random manner, Kutschera behaves like Jesus on Llull's moral tree, pruning out whatever idea appears wrongheaded to him as corrupted by religious myth on the one hand, and reducing the history of biology to a straight, progressive path towards what we, or rather he, considers to be true today.

Why should all this matter? Well, first of all, because demands on empirical accuracy and objectivity are as exacting in the humanities as they are in the sciences. It may matter whether it was indeed the oak tree - or not perhaps the date-palm, the only plant known to antiquity to possess sexuality [90] - that ranked below animals in the scale of nature (I could not find any reference to oak trees in the paper by William Bynum that Kutschera quotes in support of his rather bold claim). But there is a deeper reason. Portraying the history of science as a perennial battle between good science and evil religious influences just leaves out too much of the middle-ground that exists between these poles. What one is usually left with are equally unsatisfactory and unappealing versions of both. The current clashes between Neo-Darwinians and Intelligent Design proponents presents the public with bad theology *and *bad science, both claiming to have found answers to everything. Science as a pursuit of knowledge about the world, raising difficult questions about things that matter deeply, gets lost in this reduced landscape of *Legoland*-caricatures of science and religion.

Figure captions:

Figure 1: "Moral tree". Woodcut from Ramon Llull, *Arbor scientie*, Lyon 1515; available at http://www.gallica.bnf.fr.

Figure 2: "Natural and logical tree". Woodcut from Ramon Llull, *Logica nova*, Valencia 1512; available at http://bvpb.mcu.es/.

Figure 3: Diagram from Charles Darwin, *On the Origin of Species*, London 1859; available at http://darwin-online.org.uk/.

*Author's response: *I am grateful for this comprehensive review of the text that helped me to improved the ms considerably. Figure 1 was reproduced from a text written by a historian of science (ref. 8), who labelled this woodcut "The tree of knowledge", but I have changed this as recommended by the referee into "The Moral Tree".

All suggestions and recommendations were incorporated into the revised text.

The references O'Malley 2009 (76) and Müller-Wille 2009 (77) were added to the text and briefly discussed.

I have studied all major works of Charles Darwin - micro-organisms are not taken into consideration by the author. His brief references to "lower organisms" are confusing, unclear and, from our modern perspective, simply wrong, because Darwin was unaware of the concept of symbiogenesis that was discovered/developed after his death (see ref. 39 for a detailed discussion of this topic).

In my view, the general conclusion that religion has prevented the progress of science and is in conflict with rational thinking is justified. I do not want to change this statement, which is based on many facts and is, moreover, corroborated by corresponding conclusions published in numerous articles and books cited at the end of the text.
